# Clinical Education Stressors in Operating Room Students: A Qualitative Study

**DOI:** 10.17533/udea.iee.v39n1e08

**Published:** 2021-03-05

**Authors:** Nahid Norouzi, Behzad Imani

**Affiliations:** 1 Student Research Committee, M.Sc. Student, Department of Operating Room, Hamadan University of Medical Sciences, Hamadan, Iran. Email: N.noroozi1992@yahoo.com University of Medical Sciences Department of Operating Room Hamadan University of Medical Sciences Hamadan Iran N.noroozi1992@yahoo.com; 2 Assistant Professor, Department of Operating Room, School of Paramedical Sciences, Hamadan University of Medical Sciences, Hamadan, Iran. Email: behzadiman@yahoo.com. Corresponding Author University of Medical Sciences School of Paramedical Sciences Hamadan University of Medical Sciences Hamadan Iran behzadiman@yahoo.com

**Keywords:** knowledge, communication, learning, students, clinical clerkship, qualitative research, conocimiento, comunicación, aprendizaje, estudiantes, prácticas clínicas, investigación cualitativa, conhecimento, comunicação, aprendizagem, estudantes, estágio clínico, pesquisa cualitativa

## Abstract

**Objective.:**

The aim of this study is to explain the stressors of the clinical environment from the perspective of operating room undergraduate students.

**Methods.:**

The present study is a qualitative study of contractual content analysis type that was conducted in 2019 at Hamadan University of Medical Sciences. In this study, 10 undergraduate operating room students were selected by purposive sampling. Semi-structured interviews were used to collect data.

**Results.:**

From the analysis of interviews, 4 main categories were extracted as the stressors of operating room students of Hamadan Paramedical School in clinical learning environment: the need to receive support from the clinical environment (Insufficient students' skills in communicating with staff, Discrimination between paramedical students and residents, and Facilities available for training), lack of practical prerequisite skills (Contradiction between training and performance, and Lack of prerequisite knowledge for clinical practice), poor supportive and communication performance related to the instructor (Insufficient support of the instructor to the students against the medical staff, Evaluation criteria for instructors, and Treatment of instructor with students in presence of others), and psychological needs (Concerns about career prospects, Lack of motivational factors, and Lack of supportive counseling)

**Conclusion.:**

The results of this study showed that Operating room students are faced with many stressors in the clinical learning environment. All stressors identified in this study affected the students’ learning in the clinical setting. Lack of support for students in the clinical environment, poor practical skills training, poor support and communication performance related to the instructor, and poor psychological support of students are the factors that cause operating room student stress in the clinical environment.

## Resumen

**Objetivo.:**

El objetivo de este estudio es explicar los factores estresantes del entorno clínico desde la perspectiva de los estudiantes de pregrado que hacen prácticas en el quirófano.

**Métodos.:**

Es un estudio cualitativo del tipo de análisis de contenido contractual que se realizó en 2019 en la Universidad de Ciencias Médicas de Hamadan (Irán). En esta investigación, se seleccionaron 10 estudiantes de pregrado mediante muestreo intencional y se utilizaron entrevistas semiestructuradas para recopilar datos.

**Resultados.:**

A partir del análisis de las entrevistas, se extrajeron cuatro categorías principales como factores estresantes de los estudiantes en entornos de aprendizaje clínico en el quirófano: 1) la necesidad de recibir apoyo del entorno clínico -habilidades insuficientes de los estudiantes para comunicarse con el personal, discriminación entre estudiantes y residentes, e disponibilidad de instalaciones para la capacitación-; 2) falta de habilidades prácticas -contradicción entre capacitación y desempeño, y falta de prerrequisitos de conocimientos para la práctica clínica-; 3) pobre apoyo y comunicación con el instructor -apoyo insuficiente del instructor a los estudiantes, falta de criterios de evaluación para los instructores y de la forma como este debe tratar a los estudiantes en presencia de otros-, y 4) necesidades psicológicas -preocupación por las perspectivas de la carrera, falta de factores motivacionales y de apoyo en el asesoramiento-.

**Conclusión.:**

Los resultados de este estudio indican que la falta de apoyo en el ambiente clínico, las pobres destrezas prácticas, el insuficiente apoyo del instructor y sicológico que se brinda a los estudiantes son los factores causantes de estrés en el ambiente de prácticas clínicas en el quirófano.

## Resumo

**Objetivo.:**

O objetivo deste estudo é explicar os fatores de estresse do ambiente clínico na perspectiva de alunos de graduação em centro cirúrgico.

**Métodos.:**

Trata-se de um estudo qualitativo do tipo análise de conteúdo contratual, realizado em 2019 na Hamadan University of Medical Sciences (Irã). Nesta pesquisa, 10 alunos de graduação foram selecionados por meio de amostragem intencional. Entrevistas semiestruturadas foram utilizadas para a coleta de dados. O método de análise de conteúdo contratual foi utilizado para determinar as questões relevantes.

**Resultados.:**

A partir da análise das entrevistas, quatro categorias principais foram extraídas como fatores de estresse de alunos em ambientes de aprendizagem clínica em sala de cirurgia: 1) a necessidade de apoio do ambiente clínico - habilidades insuficientes dos alunos para se comunicarem com a equipe, discriminação entre alunos e residentes, e disponibilidade de instalações de treinamento-, 2) falta de habilidades práticas -contradição entre treinamento e desempenho, e falta de pré-requisitos de conhecimento para a prática clínica-, 3) suporte e sua comunicação do relacionado com o instrutor - suporte insuficiente do instrutor para alunos, critérios de avaliação para instrutores e como que o instrutor trata os alunos na presença de outras pessoas - e 4) necessidades psicológicas - preocupação com as perspectivas de carreira, falta de fatores motivacionais e falta de apoio na assessoria.

**Conclusão.:**

Os fatores de estresse são importantes na educação clínica desses alunos, mas, reconhecendo as causas subjacentes, se pode tomar decisões necessárias para modificar as mesmas.

## Introduction

The clinical environment is widely accepted as a key place for nursing and paramedical students to learn.(1) Clinical learning environment is an integral part of education program, accounting for half of the curriculum; because in these conditions, the student can combine and use a large volume of learned content.(2) The operating room ward is one of the medical wards of the hospital, which is known as one of the riskiest wards of the hospital due to organizational, educational, environmental, and technological needs, where the most dangerous procedures are performed.(3) Researchers have argued that the operating room environment is an important area for educating surgical technologists,(4) where the student must learn to maintain patient safety, develop clinical skills related to pre-, intra- and post-operative care create coordination and integration between their theoretical and practical knowledge, and teamwork skills in all critical and non-critical situations in such a stressful environment.(5)

Learning and adapting to different types of skills and work roles in the operating room environment is difficult; since students have to be trained in many interventions in the surgical process before, during and after surgery. However, studies show that graduates lack the skills needed to perform clinical skills.(6) In addition, there seem to be issues that prevent students from learning effectively; because students cannot perform what they learn in practice. Even students who are fully aware of the theory are likely to have problems at the client's bedside and are unable to provide care and perform skills independently.(7) On the other hand, stress is an integral part of human life that human beings face frequently today.(8) Stress is a force and a stressor is something that applies stress to an individual. Many studies have shown that stressors have a negative effect on individual’s performance and health. There are at least three distinct stressors in the field of education; the learning expected, learning environment, and the instructor.(9)

Operating room students are also affected by various stresses of clinical education, that mostly contextual and environmental factors cause this stress.(10) Experience of stress and tension can have negative effects on students' learning and clinical success and overshadow on academic performance and lead to physical and psychological disorders.(11) Undoubtedly, recognizing stressors is the first step to reduce them, and one of the best and most reliable sources to study these factors are the students themselves since they have a direct presence and interaction with the clinical education process. Awareness of stressful sources, limiting them, or raising the level of scientific and professional awareness of students makes it possible to increase their adaptation to different situations and provide a suitable environment for clinical training.(12)

Since the mental and physical health of individual students significantly influences the construction and growth of the country, the need for research on stress and identifying related factors among student communities is of particular importance. Thus, the researcher, based on her clinical and educational experiences, decided to conduct the present study to get a deeper and more comprehensive view on operating room students’ perception of clinical education with a qualitative approach aimed at explaining the stressors of clinical education from the perspective of undergraduate students in the operating room of Hamadan University of Medical Sciences in 2019.

## Methods

Study setting. The present study was a qualitative study using a contractual content analysis approach performed at Hamadan University of Medical Sciences, Hamadan, Iran.

Participants. Purposive sampling method was used for sampling students. 10 undergraduate operating room students were selected to review the data. In order to obtain a wide range of experiences and perspectives, both sexes were selected, 5 of which were male and 5 female and the age of the participants in the present study was between 19 and 25 years. Inclusion criteria included students who had at least one semester of clinical work experience and also were willing to participate in the study. Exclusion criteria were unwillingness to continue the participation in research for any reason.

Ethical considerations. The present study was conducted after receiving permission (IR.UMSHA.REC.1398.860) from the ethics committee of Hamadan University of Medical Sciences, Hamadan, Iran. Written informed consent was obtained from the participants to participate in the study. Anonymity, confidentiality of information, and the right to withdraw were considered during the study.

Data collection. Semi-structured interviews were used for data collection. The interviews were conducted individually and in the student internship area, in a quiet and peaceful place, which was desired by the participants, in a period of 20-40 minutes. The interview was recorded with the permission of the participants. To guide the interview direction toward the phenomenon under study, the researcher asked questions such as: What comes to your mind when you hear the word stress in the clinical environment? Have you ever had an unpleasant experience of stress in a clinical setting? In addition, exploration questions were used, such: Can you explain more? What do you mean? The interviews were transcribed with Microsoft Word software and prepared for analysis. During the study, numbers (P1, P2, P3, etc.) were used instead of the names of the participants. Interviews were conducted with 10 participants until data saturation.

Data analysis. In this study, the data were analyzed by the methodology of Granheim and Landman. In the first step, the text of the interviews was transcribed and used as the main research data. In the second step, the text was divided into semantic units. In the third step, abstract semantic units design and code selection were performed. According to the participants' experiences, overt and covert concepts were identified in the form of sentences or paragraphs of their words and signifying codes, followed by coding and summarizing. In the fourth step, based on the constant comparison of similarities, differences and proportions, the codes that indicated a single subject were placed in a category and subcategories and categories were classified and key codes were formed. Ambiguous points were reviewed by participants in subsequent interviews, in such a way that the points of ambiguity were removed and the position of the codes in each category was completely determined. In the fifth step at the interpretive level, the summary and central themes of each category were identified and the primary and abstract concepts were extracted.

Rigor. Four criteria of validity, credibility, reliability and transferability were used for the research rigor of Lincoln & Guba. For reliability of research, the researcher used Triangulation methods, prolong data engagement, member check, and persistent observation. For validity, the researcher tried to guarantee the validity of this research by preserving the documents related to different stages of research and reviewed by the supervisor. The research credibility was provided by such measures as participants review. For transferability, sufficient and detailed descriptions as well as the maximum variety were used.

## Results

The contractual content analysis of data revealed four main themes: Need to receive support from the clinical environment, Lack of practical prerequisite skills, Poor supportive and communication performance related to the instructor, Psychological needs.

### Theme 1. Need to receive support from the clinical environment

According to the students, having a suitable environment in which they can use all their mental and physical strength to gain new experiences is a prerequisite for effective education. This theme is very general and covers a wide range of dimensions, such as students' inadequate skills in communicating with staff in the internship environment, facilities available for training in the operating room environment, discrimination between paramedical students and surgical residents.

Insufficient students' skills in communicating with staff. Students need communication and learning from staff to gain positive learning experiences. Lack of proper communication with staff is one of the main obstacles to clinical learning and inappropriate cooperation, and communication between staff and students is one of the problematic issues in clinical education. In this regard, a participant stated the following: *... Operating room staff are not justified in how to treat the student; the student is the one who is there to learn and is not going to take their place, and if they teach something to us, it will be added to our knowledge and this field. They do not teach the student anything for any reason and establishing communication with them is not easy...* (Male student / 4th semester).

Discrimination between paramedical students and surgical residents. Lack of equipment and facilities in line with the acquisition of clinical skills in teaching centers for different groups of students were expressed in the form of phrases like: *undesirable dressing rooms and wardrobes for students in hospitals, lack of a place for conference and scientific discussion with clinical instructors, the need to communicate the same on the part of the personnel of departments with all students, whether medical or non-medical, not treating medical and non-medical students equally concerning the requirement to observe appearance criteria and differences in the quality of welfare and educational services, and respected in interactions. These are the most common form of discrimination between nursing and medical students. In this regard, a participant stated the following: ... When a surgical resident unsterilized several pairs of gloves, no one warns him, but if the operating room students do this, the whole operating room will ruin him/her, telling: you do not know, don’t come to the operation in such embargo conditions when we do not have extra gloves for you. Why do they discriminate between us??* ..." (Male student / 8th semester).

Facilities available for clinical training. Clinical education status of students requires the provision of facilities and equipment in the clinical environment and the development of educational space, which can be useful in improving the clinical education situation. For learning the minimum requirements, resources and facilities such as reference books, moulage, and student conferences during internships should be provided for the students. In this regard, a participant stated the following: ...*We do not have a conference hall in internship site, in the mornings when we come, our instructor gathers us in the room to give a brief explanation about the surgery and we have to stand for half an hour until the conference is over and we only understand the first 10 minutes. In the remaining of the sessions, we are under pressure, we are thinking of finding a place to sit, or our legs are tired .*.. (Female student / 6^th^ semester).

### Theme 2. Lack of practical prerequisite skills

This theme also includes sub-themes including lack of prerequisite practical knowledge and contradiction between theory training and performance and routine in hospital.

Lack of prerequisite knowledge. Inference from the participants' experiences shows that the existence of theoretical knowledge, in addition to providing a learning environment, acts as a determining factor in students' attitudes toward instructors, and the lack of prerequisite practical knowledge slows down the teaching-learning process and leads to formation of conflict between the instructor and the student. In this regard, a participant stated the following: *The gap between theory and practice should be small; when we read a surgical technique in the 3rd semester and now, we practically learn it in to the 5th semester in our internship, we do not remember anything*. (Male student / 6^th^ semester).

Contradiction between theory training and performance and routine in the hospital. The gap between theory and practice has side effects on students; they cannot adapt to the situation due to the conflicts between the expectations of professors and the realities of the workplace, and undesirable physical and psychological problems occur, including feelings of disability, depression, anxiety, lack of security due to inefficiency in the workplace and eventually leaving the profession. In this regard, a participant stated the following: *What we read in the textbooks are very different from what we see in the internship, and that makes us unaware of doing something, and when something is assigned to us, we get a lot of stress to do it, and the staff thinks that we do not know how to work* (Female student / 8^th^ semester).

Lack of prerequisite knowledge. Inference from the participants' experiences shows that the existence of theoretical knowledge, in addition to providing a learning environment, acts as a determining factor in students' attitudes toward instructors, and the lack of prerequisite practical knowledge slows down the teaching-learning process and leads to formation of conflict between the instructor and the student. In this regard, a participant stated the following: *The gap between theory and practice should be small; when we read a surgical technique in the 3rd semester and now, we practically learn it in to the 5th semester in our internship, we do not remember anything*. (Male student / 6^th^ semester).

### Theme 3. Poor supportive and communication performance related to the instructor

According to this theme, some instructors convey confidence and support to students and others convey stress and tension. This means that in addition to environmental factors and factors related to student readiness, factors related to the instructor significantly and even more than the previous two items affect the effectiveness of clinical education and student stress. This theme covers several sub-themes such as insufficient support of instructors to students against the medical staff, Evaluation criteria for instructors, Treatment of instructor with students in presence of others.

Insufficient support of instructors to students against the medical staff. When students are engaged in clinical work, they are confronted with operating room staff and other members of the health team who evaluate or critique their work. In this atmosphere, the friendly and supportive behavior of the clinical instructor is of special importance for the student. The received support from the instructor for student against the treatment staff increases the student's self-confidence, learning motivation, professional development, and positive outcomes. In this regard, a participant stated the following: *If we have a problem here, the instructor cannot support. The instructor is just someone who tells us a series of things but has no power in the operating room and cannot support us in front of the staff* (Male Student/ 8^th^ semester).

Evaluation criteria for instructors. Judging students' achievement of internship goals has been a challenging issue. Students have considered evaluation by clinical instructors as one of the most important problems experienced in working with clinical instructors. Problems in the field of clinical evaluation manifest in the form of student complaints, reported differences in clinical evaluation, and numerous meetings between students and nursing instructors to discuss the issue. In this regard, a participant stated the following: *The instructor only cares about washing hands. He says in the room: Did you scrub or not? But in an operation room where there are several residents and assistants and there is no place for us, what the value of washing our hands and standing on the corner is? If we are circulator, it would be much useful. However, the evaluation criterion is scrubbing. Quality is not important for the university*" (Male student / 8^th^ semester).

Treatment of instructor with students in presence of others. The friendly and respectful relationship between the instructor and the student makes the student interested in clinical learning and attending the clinic. While the ill-tempered instructor, disrespect, forcing the student to do unpleasant deeds, and unnecessary objections of the student disrupt the relationship between the instructor and the student, and the student feels that his biggest supporter is standing against him. In this regard, a participant stated the following: ... *When the instructor comes to us and commands us in presence of others, telling doing that and not doing this, this is bad, this is good, this is stressful for us*… (Female student/ 8^th^ semester).

### Theme 4. Psychological needs

The present study shows that operating room students experience various tensions in their training course that may be accompanied by psychological reactions such as depression, anxiety and stress. This theme covers 3 sub-themes includes: Concerns about career prospects, Lack of motivational factors, Lack of supportive counseling.

Concerns about career prospects. The reasons for negative attitudes and unwillingness to work in the nursing and paramedical professions include lack of a clear job description, lack of specific criteria for promotion to higher positions, dominance of physicians everywhere, lack of professional independence, and low salaries. In this regard, a participant stated the following: ...*When we see the behavior of the surgical team with the operating room staff, how they are humiliated due to a small mistake, we think about our future as the operating room staff does not have a value, and this worries us about the future of our job* ... (Male student / 2^nd^ semester).

Lack of motivational factors. In the educational program, in addition to scientific, practical and professional education, attention should be paid to improving the mental health of students so that they can play their professional roles well in the future. *...When we see the top-down behavior of surgeons with the staff, our opinion about our field is changed, that we are always under control, and we think that we should take the entrance exam from the beginning and change our field of study...* (Female student/ 8^th^ semester).

Lack of supportive counseling. Early recognition of stress and its factors as well as stress management can reduce the incidence of psychological problems, and by expanding counseling programs and prioritizing the problems of operating room students at different levels of education, it is possible to help maintain and improve their health. In this regard, a participant stated: *... When we have a problem and we are not supported, we do not have the motivation to come or work in the internship for a few days and no one asks us what the problem is ...* (Male student/ 8^th^ semester).

## Discussion

The present study expresses the objective experiences of the research participants. In the qualitative research, the findings of the present study showed a lack of support for the clinical environment, (insufficient skills of students in communicating with staff - discrimination between paramedical students and residents - poor available facilities for education), lack of practical prerequisite skills (contradiction between theory training and performance - lack of knowledge required for clinical practice), poor supportive and communicative performance of instructors (insufficient support of the instructor to students against the treatment staff - evaluation criteria for instructors and the instructor treatment with students in presence of others) and psychological needs (concern about career prospect - lack of motivational factors and lack of supportive counseling) as the stressors of operating room students of Hamadan Paramedical School in clinical learning environment.

In the case of the first primary theme, the lack of support from the clinical environment, one of the main factors affecting the students' clinical learning environment was the students' relationship with the operating room staff. Students believe that practice along with the support of nursing staff and educators in their clinical education, leads to their better education.(13) The research findings indicate that misbehavior between staff and students negatively affects the process of clinical education.(14) Many students in this study complained of staff discrimination between them and surgical residents. The results of a study conducted by Mohebi et al. showed that a high percentage of nursing students reported discrimination between them and students in other fields.(15)

Regarding the second primary theme, the contradiction between teaching and practice, it is said that at present there is no connection between what is taught to students in the classroom and what happens in the clinical environment.(16) The gap between theory and practice has side effects on students; due to the conflicts between the expectations of professors and the realities of the workplace, they cannot adapt to the situation and undesirable problems occur in them physically and mentally.(17). The studies carried out show that the disconnection of theory and practice, in addition to learning and educational problems, causes stress and do not received good support in students as well.(18) Regarding the third primary theme, the characteristics and skills of the instructor also largely determine the effectiveness of clinical training. Inappropriate interaction between instructor and student and lack of proper communication between them can probably affect all stressors in the clinical environment.(19) Given that the clinical instructors play the basic role in controlling stress, motivating and supporting students in the clinical learning environment, they should know that appropriate treatment and adequate support for the student is an important factor in creating his/her interest in the learning environment, promoting clinical skills and reducing stress, and making clinical experiences enjoyable for them.(20-22) Another controversial issue is student evaluation. Judging students' achievement of internship goals has been a challenging and stressful issue, and because this has always been one of the most important roles of nursing educators, they have always been concerned about whether their decision to evaluate a student is reflects the reality of the student's clinical performance or not.(19)

In relation to the fourth primary theme, motivation is one of the basic factors in teaching and learning that can affect students' performance in educational and research environments in different ways.(23) To have motivated and successful students in the field of education as well as graduates with appropriate skills for employment after graduation, it is necessary for decision makers through appropriate planning to reduce existing concerns about the career prospects and thus increase motivation. Consequently, wasting human and spiritual capital of the country is avoided. In particular, the goal of medical sciences universities is to train health care providers who will influence the growth and development of the country with committed and efficient activities in the health system and as a result, the promotion of public health.(24) In recent years, students' attitudes toward their future careers have become more negative, which can have many detrimental effects. Ribeiro et al., for example, cite poor future careers as a reason for elite students to emigrate; Therefore, creating suitable job opportunities, job security, teaching entrepreneurial skills by specialized professors in each field of study and creating a career counseling system in universities can make students' attitudes toward their future careers more positive.(25)

From the students' opinions, it can be concluded that education and health managers at higher levels should pay attention to weaknesses, because most of the problems and stressors raised can be modified by management measures. It is hoped that by addressing issues such as environmental problems and lack of facilities, poor performance of instructors, the gap between theoretical and clinical education, lack of supportive counseling, the roots of the problems will be identified so that students can act competently in the clinical environments in the field of effective patient care. It is suggested that the stressors are identified, the findings of the present study are taken into account, and planning is done to solve problems and improve the conditions of clinical education. These measures should be put at the top of paramedical educational programs in order to take steps towards community health. It is hoped that applying the results of this study by clinical teachers and students will help improve the clinical education process. Further studies in relation to each of the categories obtained in the present study are recommended.

Conclusion. The results showed that not supporting students in the clinical environment, poor practical skills training, poor supportive and communicative performance of the instructor, and not supporting the psychological needs of students are among the stressors of the students' clinical environment. The use of the main classes of stressors of the clinical educational environment designed in this study can help educators and educational administrators to use strategies to strengthen the clinical education of operating room students and subsequently improve their learning.
